# Monitoring Tritrophic Biocontrol Interactions Between *Bacillus* spp., *Fusarium oxysporum* f. sp. *cubense*, Tropical Race 4, and Banana Plants *in vivo* Based on Fluorescent Transformation System

**DOI:** 10.3389/fmicb.2021.754918

**Published:** 2021-10-13

**Authors:** Ping He, Shu Li, Shengtao Xu, Huacai Fan, Yongfen Wang, Wei Zhou, Gang Fu, Guangyu Han, Yun-Yue Wang, Si-Jun Zheng

**Affiliations:** ^1^State Key Laboratory for Conservation and Utilization of Bio-Resources in Yunnan, Ministry of Education Key Laboratory of Agriculture Biodiversity for Plant Disease Management, College of Plant Protection, Yunnan Agricultural University, Kunming, China; ^2^Yunnan Key Laboratory of Green Prevention and Control of Agricultural Transboundary Pests, Agricultural Environment and Resources Institute, Yunnan Academy of Agricultural Sciences, Kunming, China; ^3^Institute of Tropical and Subtropical Industry Crops, Yunnan Academy of Agricultural Sciences, Baoshan, China; ^4^Biotechnology Research Institute, Guangxi Academy of Agricultural Sciences, Nanning, China; ^5^Institute of Plant Protection, Guangxi Academy of Agricultural Sciences, Nanning, China; ^6^Bioversity International, Kunming, China

**Keywords:** *Bacillus* spp., biocontrol, electro-transformation, RFP-labeled *Bacillus*, *Bacillus* interaction with TR4

## Abstract

*Bacillus* spp. is effective biocontrol agents for Fusarium wilt of banana (FWB), tropical race 4 (TR4). This study explores the colonization by *Bacillus subtilis*, *Bacillus velezensis*, and *Bacillus amyloliquefaciens* of host banana plants and elucidates the mechanism of antagonistic TR4 biocontrol. The authors selected one *B. subtilis* strain, three *B. velezensis* strains, and three *B. amyloliquefaciens* strains that are proven to significantly inhibit TR4 *in vitro*, optimized the genetic transformation conditions and explored their colonization process in banana plants. The results showed that we successfully constructed an optimized fluorescent electro-transformation system (OD_600_ of bacteria concentration=0.7, plasmid concentration=50ng/μl, plasmid volume=2μl, transformation voltage=1.8kV, and transformation capacitance=400Ω) of TR4-inhibitory *Bacillus* spp. strains. The red fluorescent protein (RFP)-labeled strains were shown to have high stability with a plasmid-retention frequency above 98%, where bacterial growth rates and TR4 inhibition are unaffected by fluorescent plasmid insertion. *In vivo* colonizing observation by Laser Scanning Confocal Microscopy (LSCM) and Scanning Electron Microscopy (SEM) showed that *Bacillus* spp. can colonize the internal cells of banana plantlets roots. Further, fluorescent observation by LSCM showed these RFP-labeled bacteria exhibit chemotaxis (chemotaxis ratio was 1.85±0.04) toward green fluorescent protein (GFP)-labeled TR4 hyphae in banana plants. We conclude that *B. subtilis*, *B. velezensis*, and *B. amyloliquefaciens* can successfully colonize banana plants and interact with TR4. Monitoring its dynamic interaction with TR4 and its biocontrol mechanism is under further study.

## Introduction

Fusarium wilt of banana (FWB) caused by *Fusarium oxysporum* f. sp. *cubense*, especially Tropical Race 4 (TR4), is one of the most destructive diseases affecting the crop (Ghag et al., 2015; Presti et al., 2015; Carvalhais et al., 2019). Pathogen spores invade the vascular bundles of banana roots through wounds and then extend to the corms and pseudostems, causing the vascular bundles to become brown and necrotic, the leaves gradually wither and eventually the whole plant dies (Swarupa et al., 2014). Due to its characteristic of surviving in the soil for decades, once the pathogen is introduced into the soil, the infected banana orchard cannot be used for growing susceptible banana cultivars, which seriously affects the sustainable development of the banana industry, as there are few proven TR4-resistant cultivars (Ploetz, 2006, 2015).

Like all the other *Foc* strains, TR4 cannot be controlled using fungicides and cannot be eradicated from soil using fumigants (ProMusa, 2021). Crop rotation and intercropping have been used to reduce the infections and inoculum levels (Nadarajah et al., 2016). In China, farmers have been growing bananas in the presence of TR4 by rotating or intercropping with Chinese leek (*Allium tuberosum*; Nadarajah et al., 2016; Li et al., 2020a,b). The most effective solution supporting continued production of bananas in infested soils would be replacing susceptible cultivars with resistant ones. However, almost all important banana cultivars are susceptible to TR4 (Ploetz, 2015; Chen et al., 2019; Sun et al., 2019), and most commercial cultivars are triploid and sterile (non-seed bearing) makes banana breeding more difficult (Hwang and Ko, 2004; Heslop-Harrison and Schwarzacher, 2007). At present, few TR4-resistant banana cultivars have been bred and popularized (Daniells, 2011). Furthermore, even those cultivars still have to adapt to local cultivation practices and conditions. As a result, the spread of TR4 has led to an increase in research on biological control and biocontrol agents (BCAs) in suppressing the pathogen (Nadarajah et al., 2016; Bubici et al., 2019; Damodaran et al., 2020).

So far, many microbes such as *Trichoderma* spp., *Pseudomonas* spp., and *Bacillus* spp. have been widely used as BCAs (Bubici et al., 2019). The characteristics of spore-forming and rapid growth of *Bacillus* species confer them with an important advantage over other beneficial biological control microorganisms. In addition, many *Bacillus* species can synthesize a large number of secondary metabolites, which play a key role in antibiosis against detrimental microorganisms (Radhakrishnan et al., 2017; Fira et al., 2018). *Bacillus subtilis* is a representative *Bacillus*, which could produce a variety of antibiotics with different structures and activities; it also exhibits a wide range of antibacterial activities against different plant pathogens under *in vitro* conditions (Stein, 2005). *Bacillus amyloliquefaciens*, a type of Gram-positive bacterium (Priest et al., 1987), highly homologous with *B. subtilis*, has a single nutrient requirement and is harmless to the environment and human health. Due to these positive characteristics, many *B. amyloliquefaciens* strains have been isolated and identified as having significant inhibition and control effects on *Ceitocybe bescens* and *Fusarium oxysporum* (Guan and Jiang, 2013). Wang et al. (2015) and Xue et al. (2015) found that *B. amyloliquefaciens* combined with organic fertilizers can significantly reduce the incidence of Fusarium wilt. The *B. amyloliquefaciens* isolated by Zhang et al. (2014) showed a good biocontrol effect on banana wilt, and this strain can produce IAA and siderophore, promote the growth of banana plants, and has a high biocontrol potential. *Bacillus velezensis* is also a Gram-positive bacterium that is closely related to *B. amyloliquefaciens*. FZB42 is currently the most researched *B. velezensis* strain, which has been commercialized, and it is effective against various pathogens caused by bacteria and fungi (Borriss et al., 2011). Fan et al. (2011) integrated a plasmid carrying a gene encoding a fluorescent protein into the chromosome of the FZB42 strain and successfully observed the colonization and distribution in the roots of corn, *Arabidopsis*, and duckweed. However, so far there are few reports on the colonization and distribution of *Bacillus* on banana plants. Understanding how these beneficial microorganisms colonize and distribute in host banana plants will provide an important and favorable theoretical basis for using biocontrol strains to control FWB.

Most biocontrol *Bacillus* species are soil microorganisms that colonize the rhizosphere of plants and directly or indirectly promote plant growth through different mechanisms (Compant et al., 2005; Scherwinski et al., 2008; Malfanova et al., 2013). These play an important role in many fields, such as ecological restoration, biocatalysis, and biological control. The successful colonization of biocontrol strains is a prerequisite for the development of biocontrol promotion and disease prevention, and it is vital to explore their interaction processes with plants (Kang, 2019). Fluorescence transformation is currently the most successful approach for studying the colonization of *Bacillus* spp. and the interaction with plants *in vivo* (He, 2014). However, many wild-type *Bacillus* species with good bio-promoting and disease-preventing effects cannot easily form competent cells because of the unknown cellular restriction-repair system, which leads to constraints to a low efficiency of electric shock transformation (Alegre et al., 2004; Yasui et al., 2009). This seriously hinders banana research on horizontal manipulation or modification of *Bacillus*, as well as the further utilization and exploitation of the potential value of these biocontrol agents. Hence, there is a need to develop a reliable and efficient fluorescent-transformation system for monitoring the interactions between *Bacillus*, *Foc* TR4, and banana plants.

**Figure 1:**
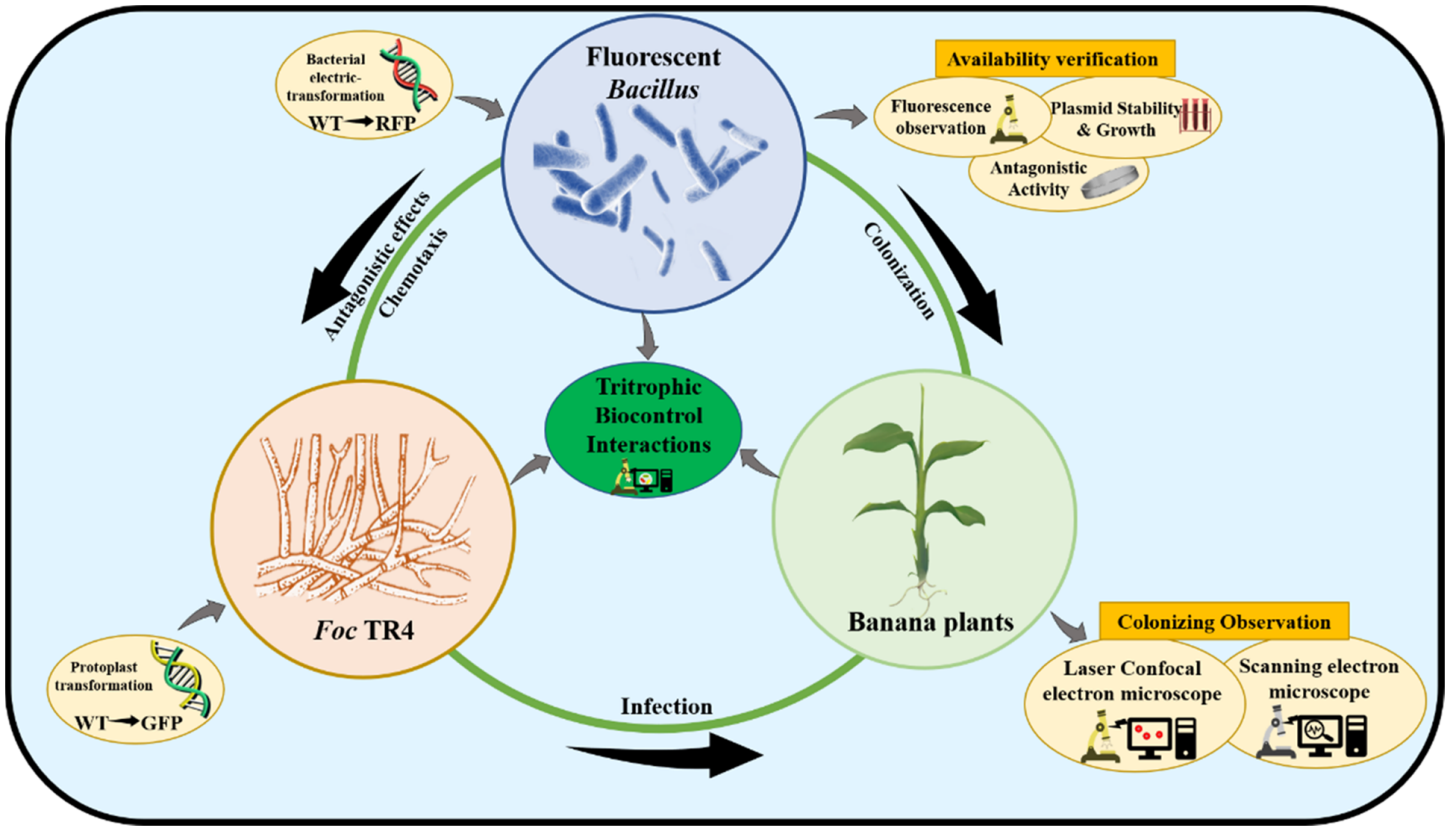
The schematic diagram of tritrophic biocontrol interactions.

In this study, seven *Bacillus* species containing one *B. subtilis*, three *B. velezensis*, and three *B. amyloliquefaciens* strains with strong antagonistic effects on TR4 *in vitro* were selected. In order to obtain stable fluorescent-marked transformants and develop an efficient genetic transformation system, pYP69 carrying red fluorescent protein (RFP) was used as the fluorescent expression vector, which was successfully introduced into wild-type *Bacillus* strains according to the optimized experimental parameters. Furthermore, laser confocal observation confirmed that the fluorescent-transformed strains could be used for monitoring how *Bacillus* colonizes host banana plants (Figure 1).

## Materials and Methods

### Source of Strains and Plasmid

*Bacillus subtilis* strain YN1419 (GenBank Accession No. MW647761) was isolated from banana cultivar Brazilian in Xishuangbanna, Yunnan, China. *Bacillus velezensis* strain YN1282-2 (GenBank Accession No. MW663765) was isolated from banana cultivar GCTCV-119. *Bacillus velezensis* N67 (GenBank Accession No. MW672323), WBN06 (GenBank Accession No. MW672324), and *B. amyloliquefaciens* G9R-3 (GenBank Accession No. MW674627; Zhou et al., 2020), HN04 (GenBank Accession No. MW674626) were isolated from Guangxi banana plantations. *Bacillus amyloliquefaciens* YN0904 (GenBank Accession No. MW647760) was isolated from Yunnan banana plantation. All experimental strains had proven strong *Foc* TR4 antagonistic effects *in vitro* (Li et al., 2021). *Foc* TR4 strain 15-1 (Zhang et al., 2018) was isolated from infected banana plants in Xishuangbanna, Yunnan, China. GFP-TR4 was constructed in our laboratory (Zhang et al., 2018) and was used in monitoring the interaction with bacteria. The plasmid pYP69 expressing RFP and the chloramphenicol-resistance gene (Supplementary Figure S1) were obtained as a gift from Dr. Yongmei Li (Plant Protection College of Yunnan Agricultural University, Kunming, China) and Dr. Yiyang Yu (Plant Protection College of Nanjing Agricultural University, Nanjing, China). pYP69 was constructed by the pYC127 as backbone and cloned with mKate2 coding sequence (Chen et al., 2012). The *Escherichia coli* DH5α was purchased from Beijing Biomed Biotechnology Co., Ltd. (Beijing, China). The strains were stored in 25% glycerol at −80°C. The isolates were reactivated on nutrition agar (NA) medium at 37°C for 24h.

### Culture Media

LB broth medium (LB; tryptone 10g, yeast extract 5g, NaCl 10g, pH 7.0 for 1l with deionized water) was used to cultivate the bacteria and prepare the bacterial suspension. Potato Dextrose Agar (PDA) medium (200g potato, 20g glucose, 20g agar, diluted to 1l, natural pH) was used to activate the *Foc* TR4 and conduct the dual-culture experiments. Growth Medium [GM; LB with 3% glycine (Gly), 1% _DL_-threonine (_DL_-Thr), 0.03% Tween 80, and 9.1% sorbitol] was used to prepare competent cells. ETM buffer (40ml glycerol, 360ml deionized water, 36.4g sorbitol, 36.4g mannitol, 0.25mM KH_2_PO_4_, 0.25mM K_2_HPO_4_, and 0.5mM MgCl_2_) was used to wash away the ion components in competent cells. Recovery Medium (RM; LB with 9.1% sorbitol and 6.92% mannitol) was used for resuspension of competent cells after electroporation.

### Detection of Plasmid pYP69 Expression in *Escherichia coli*

The *Escherichia coli* competent cells were thawed on ice. One microliter pYP69 was mixed gently into competent cells and put it on ice for 30min stationary Then, the heat shock was applied at 42°C for 60s, and then, the culture was put on ice for 2min. Five hundred microliter LB liquid medium was then added to the culture and resuscitated at 37°C, swirling at 180rpm for 60min, and then, the transformative culture was evenly spread on the 100μg/ml ampicillin-resistant medium. The transformative plates were cultured at 37°C for 16h, and then, single colonies were selected to observe fluorescence by the Fluorescence Microscope (Nikon 80I).

### Plasmid Extraction

The *E. coli*-carrying pYP69 plasmid was inoculated into LB liquid medium containing ampicillin (100μg/ml), and cultured at 37°C, swirling at 220rpm for 16h. Plasmid extraction followed the instructions accompanying the OMEGA Plasmid DNA Extraction Kit (E.Z.N. A® Plasmid DNA Mini Kit I).

### Electroporation

#### Preparation of Competent Cells

Competent cells preparation (Zhang et al., 2011) was carried out as follows. *Bacillus* strains stored at −80°C were inoculated in the LB solid medium overnight. Each single colony was individually selected and inoculated in 30ml GM liquid medium by inoculating loop, cultivated at 37°C, 220rpm for 16h. Two milliliter cultured bacteria suspension was inoculated into 200ml GM liquid medium, cultured at 37°C, and swirled at 220rpm for 3h. OD_600_ value was determined by spectrophotometer (Nanophotometer NP80 Touch). When OD_600_ value reached 0.5, 3% Gly, 1% _DL_-Thr, and 0.03% Tween 80 were added into the culture for cell-wall weakening. The culture was arrested when the OD_600_ value reached 0.7. The bacterial suspension was put on ice for 10min and centrifuged at 4°C, 1,300*g* for 10min; then, 50ml precooled ETM solution was used to wash away the ion components in the GM medium for three times. About 500μl ETM was used to suspend the competent cells, competent cells were divided into 100μl per EP tubes and stored at −80°C.

#### Electroporation

The transformation (Kang, 2019) was carried out as follows. Two microliter plasmid pYP69 were added into 100μl competent cells culture, mixed the plasmid and competent cells, and electroporation was carried out. The electroporation voltage was 1.8kV, and the capacitance was 400Ω. After electroporation, 1ml RM medium was immediately added to the electroporation cup and then transferred to a 1.5ml centrifuge tube, cultured at 37°C, shook at 220rpm for 4h. The culture was spread on the LB solid plate containing 10μg/ml chloramphenicol, the positive transformants designated according to the format of RFP-*Bacillus*, e.g., RFP-N67, etc., were selected, and the fluorescence labeling was observed under the fluorescence microscope (Excitation wavelength: 555nm/Emission wavelength: 584nm).

### Stability Determination of Plasmid pYP69 in *Bacillus*

The activated RFP-*Bacillus* strains were inoculated into liquid LB medium without antibiotics, cultured at 37°C, and shook at 180rpm, with samples being taken every 5h and spread on LB solid plates, and bacteria colonies were observed by fluorescence microscope. The percentage of red fluorescent colonies in total cells colonies used to calculate the stability of the plasmid in the RFP-strains. Three replicates were conducted.

### Cell Growth Determination of RFP-Strains

Red fluorescent protein-*Bacillus* and wild-type (WT) strains were inoculated in the LB liquid medium and cultured to the concentration was OD_600_ reaching 1.0, and then, the bacteria suspension was transferred to the blank medium at a ratio of 1%. Bacteria were cultured at 37°C and shook at 180rpm for 60h. OD_600_ values of samples were measured every hour in the first 6h. OD_600_ was measured every 2h during 8–20thh, and OD_600_ was measured every 4h during 22nd–60thh. Three replicates were made.

### Antagonistic Activity of RFP-Strains on TR4

The dual-culture method was used to compare the antagonistic activity of the RFP-labeled *Bacillus* and WT strains against *Foc* TR4. TR4 was activated in PDA for 7days at 28°C, and RFP-labeled bacteria and WT bacteria were activated at 37°C for 24h. Individual 5mm diameter disks of TR4 hyphae were placed in the center of each PDA plate. Plates were then inoculated with the RFP strain and the WT strain at 2.5cm from the center by using inoculating loop. These were cultivated for 7days, and the growth of TR4 hyphae was measured. The inhibition dual culture assay was carried out in three replicates.

### Colonizing Observation of Biological Control *Bacillus*

A 10ml red fluorescent bacterial suspension (cultivated 24h at 37°C and 180rpm, diluted to 1×10^6^cfu/ml) was pour into the tissue-culture bottle which cultured five banana plantlets by MS medium, gently shaking the bottle and placing in a culture incubator (30°C, 80% humidity, 12h light/12h dark), with the treatment adding just sterilized water as the control. Five replicates were made. Ten banana plant roots per treatment were randomly selected after inoculation for 7days, and the roots were washed with flowing sterile water to remove the medium.

For fluorescent microscopy, tissue slices (thickness: 50nm) were excised by freezing microtome and any bacteria in root tissues were observed by Laser Confocal Electron Microscope (Leica TCS-SP8). Excitation/emission wavelengths were 561nm/570–640nm for RFP (mKate2 protein).

For scanning microscopy, the critical point drying sample processing method was conducted. Tissue slices (thickness: 50nm) were immersed in FAA fixative (5ml 38% formaldehyde, 5ml glacial acetic acid, 90ml 70% ethyl alcohol, and 5ml glycerol) at 4°C overnight. Then: the fixed samples were immersed in 50, 60, 70, 80, 90, and 95% alcohol successively to dehydrate 30min was conducted in each alcohol concentration, then the samples were immersed in absolute ethanol for 30min, and three replicates were conducted. Banana tissue samples were then put into Critical Point Dryer (K850) and Cressington Sputter Coater (108 AUTO) to spray gold coating. The fixed samples were put into Scanning Electron Microscope (ZEISS Sigma 300, Germany) to observe the bacterial colonization.

### Laser Confocal Electron Microscope Observation of Interaction *in vivo*

In order to observe the interactions between strains and TR4 *in vivo*, 3ml RFP-labeled bacterial culture in 9% physiological saline solution was inoculated into the leaf vascular bundles of the banana cultivar Brazilian by injection (Liu et al., 2020), and a 5mm agar disk with GFP-labeled TR4 mycelia (Zhang et al., 2018) was placed on the wound by inoculation loop. The latest fully expanded leaves were selected. Fluorescent observation by laser scanning confocal microscope (Leica TCS-SP8) was carried out after the leaves were cultured in 28 °C, 60% light, and 50% humidity for 7days. Excitation/emission wavelengths were 561nm/570–640nm for RFP (mKate2 protein). Excitation/emission wavelengths were 488nm/500–540nm for GFP (AmCyan protein).

### Chemotaxis Assay of Bacteria to Pathogen

Seven day-old *Foc* TR4 were inoculated in 50ml diluted PDB (1: 50; v: v in H_2_O) at 28°C with shaking at 150rpm for 24h to obtain spores suspension of *Foc* TR4. Suspension was then washed twice with sterile ddH_2_O and incubated for 48h in 5ml sterile ddH_2_O at 28°C and 150rpm. The supernatant was sterilized by filtration through a 0.22μm membrane (Millex-GP) for use.

To obtain bacterial suspension, 1×10^7^ colony ml^−1^ RFP-N67 strains were grown in LB overnight at 37 °C, washed either with sterile ddH_2_O, and diluted in ddH_2_O to the OD_600_ of 0.1.

Chemotaxis capillary assays (Palmieri et al., 2020) were carried out as follows. 250μl bacterial suspension was added to the well (1.5cm×1.5cm) in the glass slide together with a 10μl capillary containing the test compound (*Foc* TR4 hyphal exudate or ddH_2_O). Slides were incubated for 60min at 28°C, capillaries were carefully lifted, the content was serially diluted and plated onto LA medium with 10μg/ml chloramphenicol, and CFUs were counted 24h after incubation at 37°C. The chemotaxis ratio was calculated by dividing the number of bacteria in the tube containing the test compound (*Foc* TR4 hyphal exudate) by the number of bacteria in the tube containing the control (ddH_2_O). All experiments included four replicates and were performed three times with similar results.

### Statistical Analysis

Data were analyzed by one-way ANOVA using the SPSS version 18.0 for Windows (Chicago, IL, United States). The figures and charts were drawn using ORIGIN 2018 (Massachusetts, United States).

## Results

### Plasmid pYP69 Expresses Fluorescence in *Escherichia coli*

**Figure 2:**
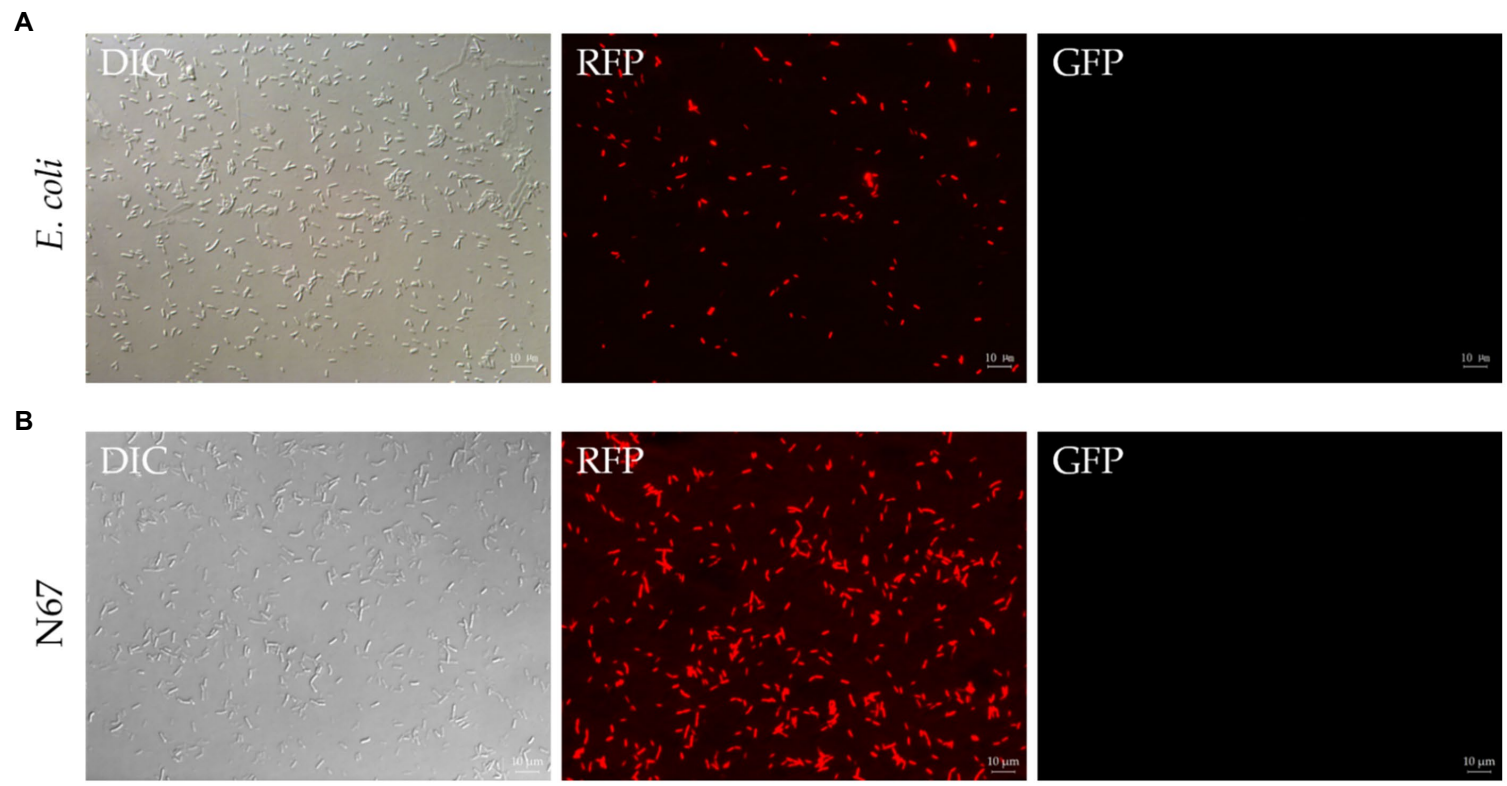
Fluorescence observation of *Escherichia coli* and N67 under microscopy. DIC, differential interference contrast field; RFP, red fluorescence field; and GFP, green fluorescence field. **(A)** Representative fluorescent micrograph of *E. coli* under fluorescent microscope. Heat-shock transformation was used to transform pYP69 into *E. coli* DH5α competent cells. The positive transformants on the solid medium containing 100μg/ml ampicillin/ could be observed under the microscope. **(B)** Representative fluorescent micrograph of N67 under fluorescent microscope. Electric-transformation was used to transform pYP69 into N67 competent cells. The positive transformants on the solid medium containing 10μg/ml chloramphenicol could be observed under the microscope. Scale bar: 10μm.

Before transforming *Bacillus*, we aimed to verify whether this plasmid could be expressed in *E. coli*, preserved, and extracted. Heat-shock transformation was used to transform the plasmid pYP69 into *E. coli* DH5α competent cells. We obtained positive transformants on plates containing ampicillin. Colonies glowed an obvious red color under the fluorescence microscope, and the red *E. coli* cells could still be visualized even after being cultured in the liquid. This verified that RFP-labeled cells can be observed under the fluorescence microscope (Figure 2A), indicating that the plasmid pYP69 can be expressed in *E. coli*.

### Plasmid pYP69 Expresses Fluorescence in *B. sublitis*, *B. velezensis*, and *B. amyloliquefaciens*

Fluorescence labeling is one of the best methods for tracing bacterial colonization (including *B. sublitis*, *B. velezensis*, and *B. amyloliquefaciens*) in plants. Here, electroporation with optimized conditions was used to transfer the plasmid pYP69 into competent cells of WT *Bacillus* strains. Positive transformants of *Bacillus* were obtained on the solid plate of 10μg/ml chloramphenicol. These can be seen with the naked eye as the colonies of strains formed on the antibiotic plate gradually become light red. Fluorescence microscope observation results showed that the plasmid pYP69 has been successfully transformed into the WT strains and can express RFP in the *Bacillus* strains (Figure 2B; Supplementary Figures S2A–E). Several repeated experiments showed that although there was still relatively low trans-formants efficiency (1×10^2^–10^3^cfu/μg of plasmid DNA); stable transformants from each *Bacillus* strain had already been generated.

### RFP-Labeled Strains Possess High Plasmid Stability

**Figure 3:**
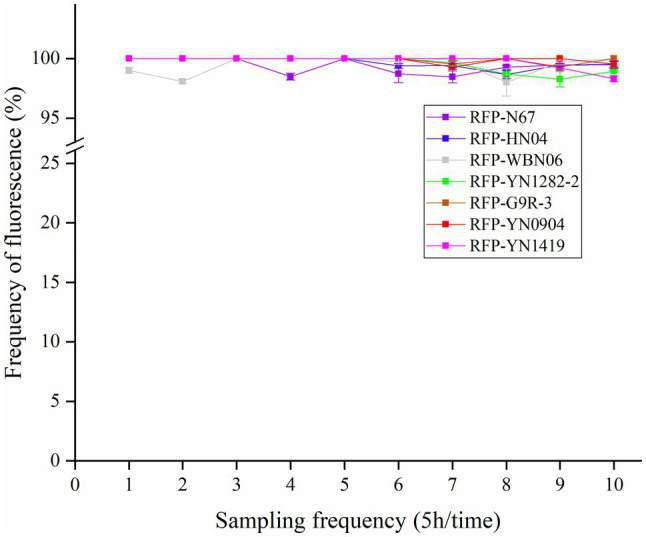
Fluorescence plasmid retention frequency of RFP-labeled *Bacillus*. Samples were taken every 5h. The proportion of colonies on a non-resistant plate with fluorescence was assessed (with three replicates) under fluorescence microscope to determine the frequency of plasmid retention.

The stability of plasmid expression in the strains is important for the construction of fluorescent strains. Under condition of no antibiotic pressure, the red fluorescent strain was cultured in serial dilutions, and the samples were taken every 5h. The dilution was evenly spread on a non-resistant plate. The proportion of colonies with fluorescence was counted under a fluorescence microscope to determine the frequency of plasmid retention. We found that the frequency of RFP plasmid cells is all greater than 98% after being cultured for 10 consecutive generations (Figure 3). Results confirmed the plasmid is rarely lost due to the proliferation of bacterial cells, indicating that the plasmid pYP69 can be stably expressed in these *Bacillus* strains, and it can be used for experiment tracing colonization and migration in plants.

### RFP-Labeled Strains Have the Similar Growth Condition of WT-Strains

**Figure 4:**
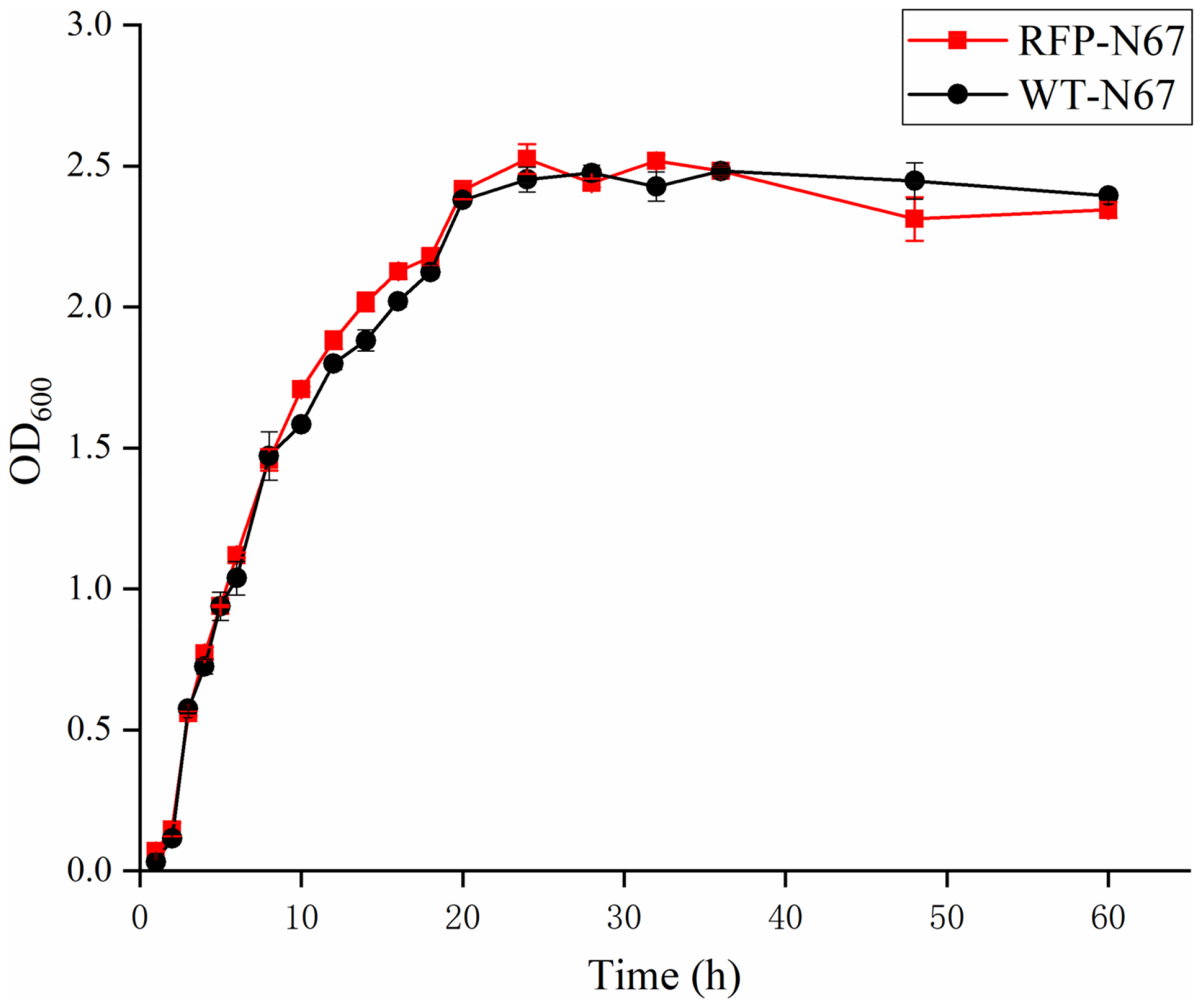
The growth rate of RFP-N67 compared to wild-type N67. OD600 measures were used to determine bacterial growth rate. Three replicates were included.

The growth of the RFP-*Bacillus* and WT strains was compared under the same inoculation and culture conditions. The results showed that the growth of RFP-YN0904, RFP-YN1282-2, RFP-WBN06, and RFP-N67 was consistent to WT strains, indicating that the plasmid pYP69 had no significant effect on the growth of these three *B. velezensis*. The wild-type strains of HN04 and G9R-3 grew slight faster in the log phase and reached the stable phase earlier than RFP strains, the wild-type strains YN1419 grew lower before the stable phase than the RFP strain, but they did not significantly affect normal growth of RFP bacteria (Figure 4; Supplementary Figures S3A–E). We speculate that it may be that the introduction of multicopy large plasmids affects the growth rate of *B. amyloliquefaciens* and *B. subtilis*, and good fluorescence performance of the plasmids in the *Bacillus* host may also create an additional metabolic burden on the strains.

### RFP-Labeled Strains Retain Their Antagonistic Activity Against TR4

**Figure 5:**
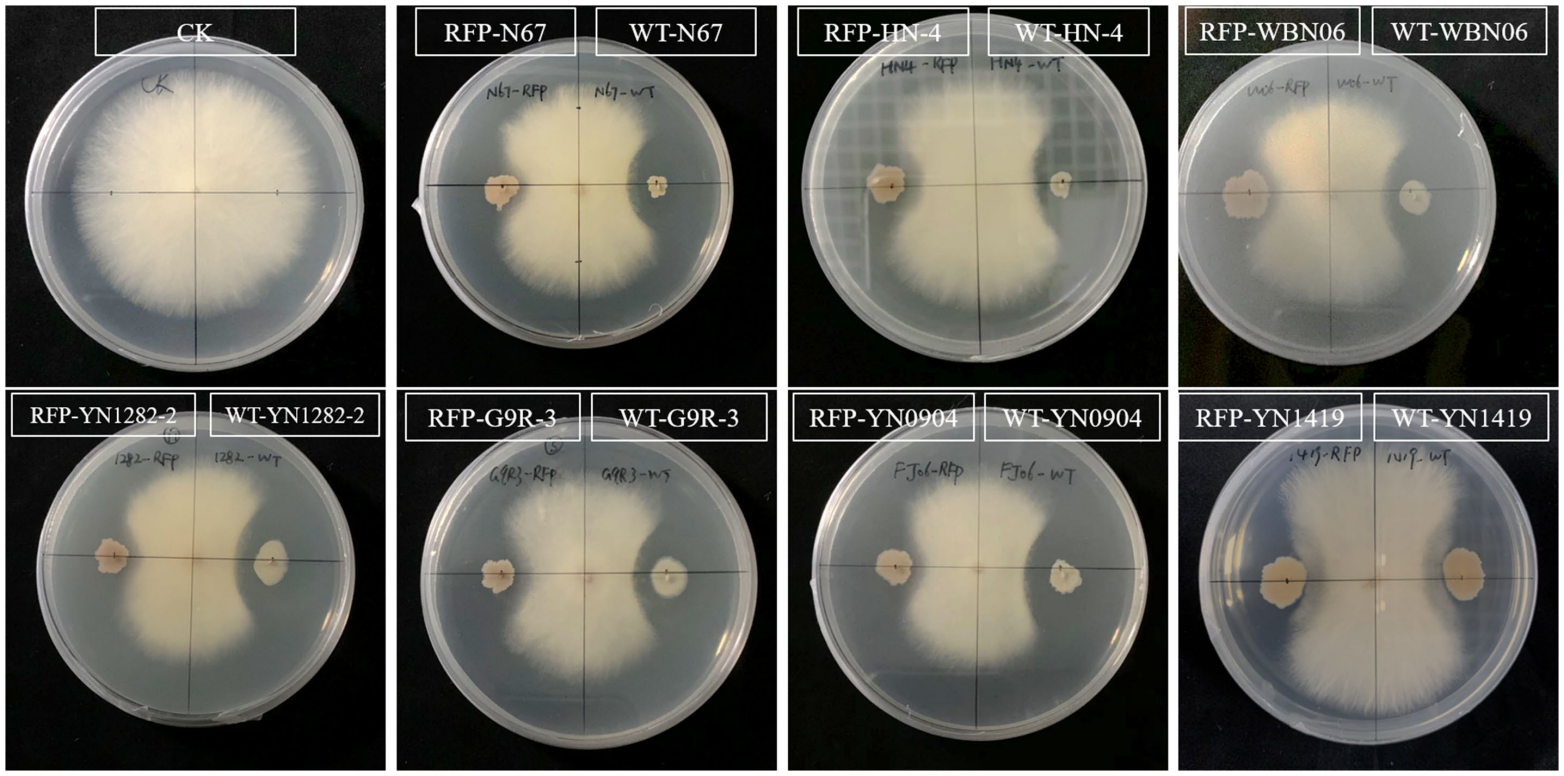
Antagonistic effect of RFP-labeled *Bacillus* compared to the WT *Bacillus*. The plate inoculated with pathogen only was used as the control (CK). The antagonistic effect of RFP-strains (left) and WT strains (right), respectively, and the pathogen was inoculated in the center. Five replicates were used.

The metabolic burden caused by the introduction of exogenous plasmids sometimes affects other functions of the strain. In order to explore the effect on the biological characteristics of plasmid pYP69 insertion, the antagonistic activities of the RFP-*Bacillus* strain and the WT strain against the pathogenic fungus *Foc* TR4 were compared. The results showed that there was no significant difference in the antagonistic activity of the two types of strains against the tested pathogenic fungi (Figure 5; Supplementary Table S1). It shows that the expression of plasmid pYP69 does not affect the inhibitory activity of the *Bacillus* strains on the growth of the pathogenic fungus.

### *Bacillus* Can Successfully Colonize Banana Root Cells

**Figure 6:**
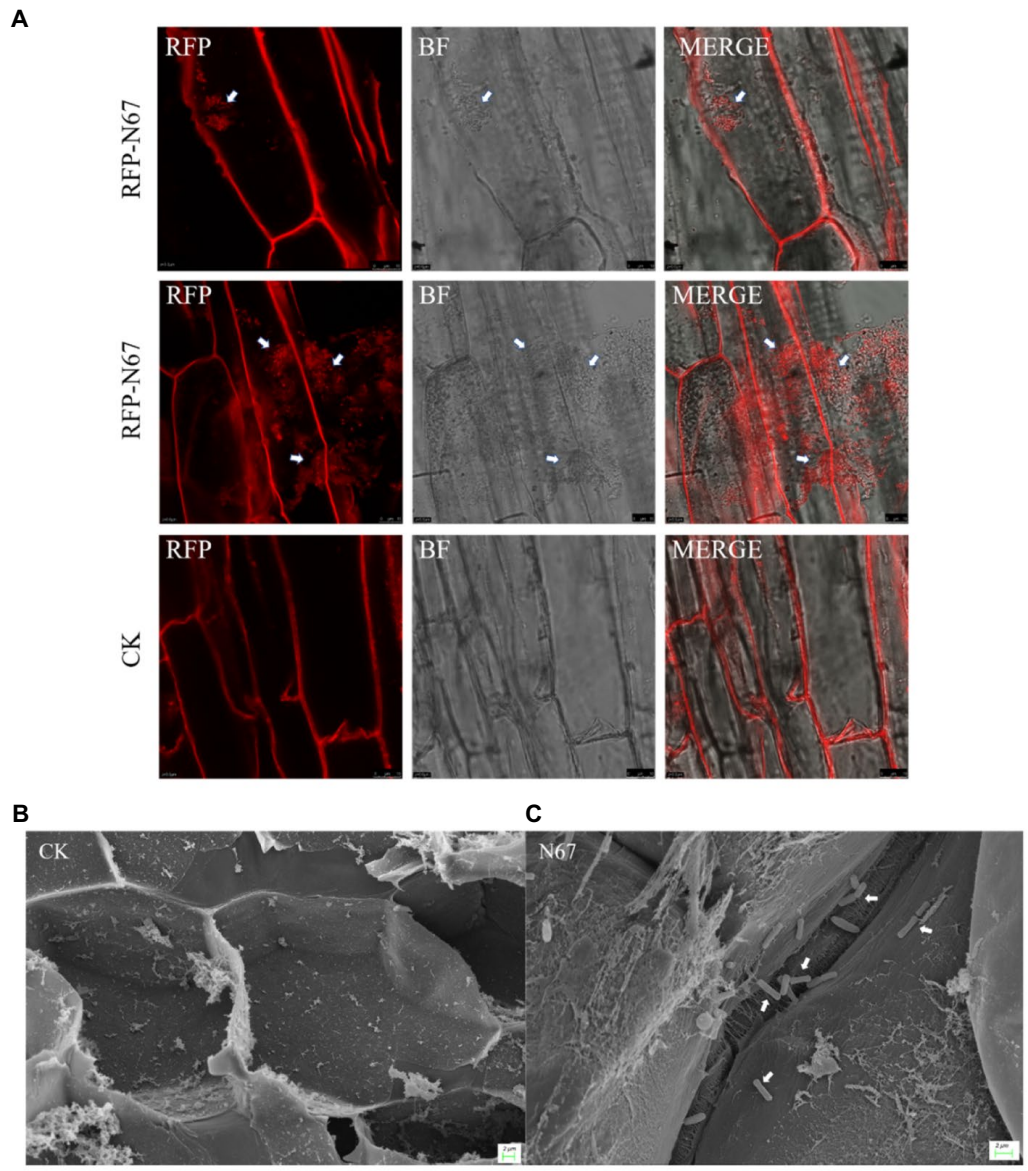
*Bacillus velezensis* N67 colonies in banana roots. Banana roots were randomly selected 7days after inoculant, and the roots were washed with sterile water to remove the medium and bacteria adhering to the surface. Tissue slices were cut out to Laser Confocal Microscopy (LCM) observation and subjected to critical point drying before Scanning Electron Microscopy (SEM) observation. **(A)** Representative fluorescent micrograph of RFP-N67 distributed in banana root cells. Scale bar: 10μm. **(B)** Scanning electron micrograph of control treatment that there were no any bacteria in the banana root cell. **(C)** Representative micrograph of RFP-N67 distributed in banana root xylem cells. The white arrows indicate the bacteria RFP-N67. Three replicates were used. Scale bar: 2μm.

Biocontrol bacteria successfully colonizing plants are a necessary pre-condition for their biocontrol function. To explore the colonization capacity of *Bacillus* in banana plants, we inoculated tissue-cultured banana plantlets with *B. velezensis* RFP-N67. After 7days of co-culture, we sampled the banana plantlets’ roots, and slice observation by Laser Confocal Microscopy (LCM) showed that RFP-N67 colonized the banana roots cells (Figure 6A) and Scanning Electron Microscopy (SEM) confirmed the bacteria could successfully colonized the roots xylem cells (Figures 6B,C). We also found that there were no bacteria in banana plants subject to low inoculation concentrations (1×10^3^–10^5^cfu/ml), and that *Bacillus* can enter the root when the inoculation concentration reached a higher density (1×10^6^cfu/ml). However, excessive concentrations could damage banana plantlets.

### *Bacillus* Exhibits Chemotaxis Toward *Foc* TR4 in Banana Plants

**Figure 7:**
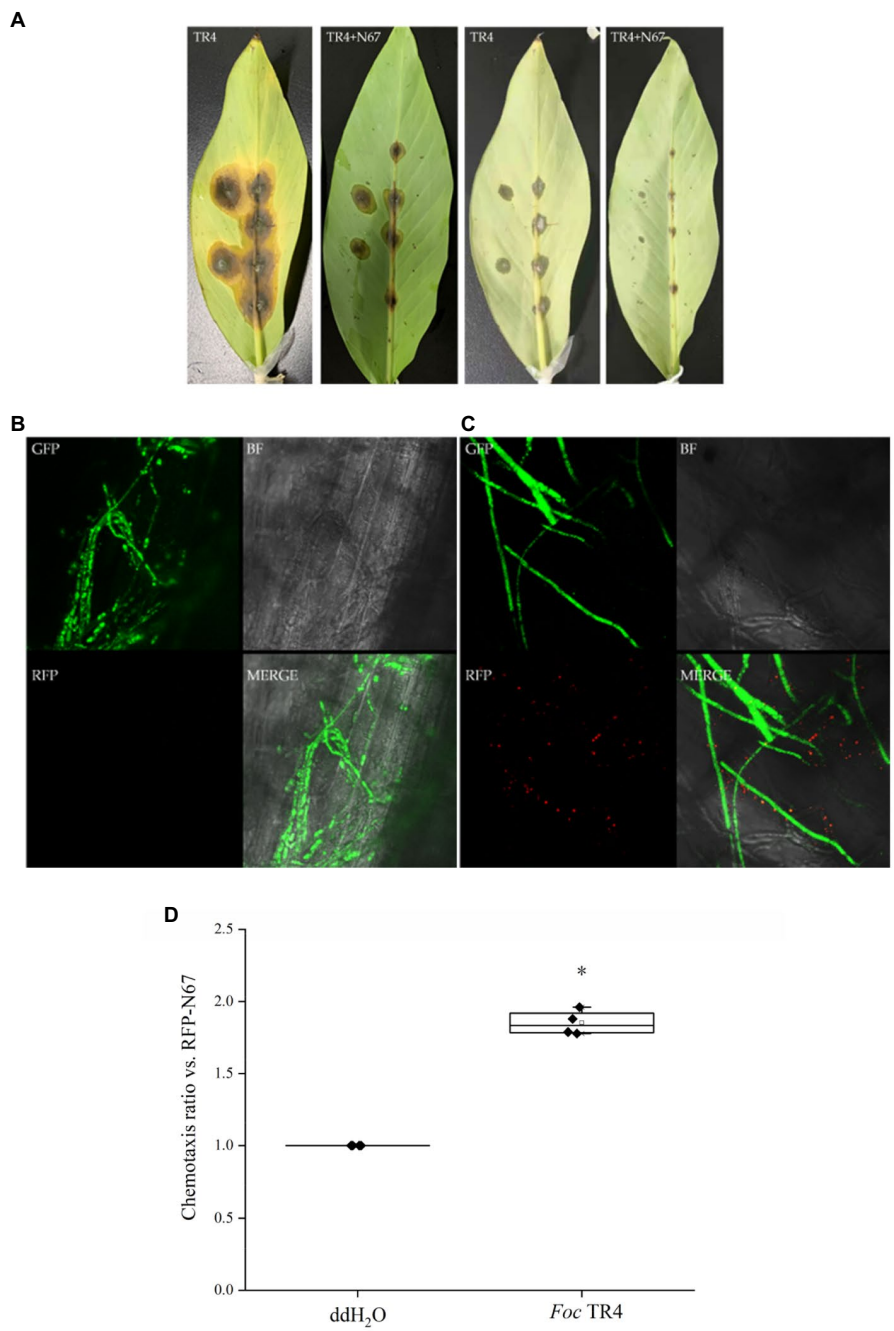
*Bacillus velezensis* RFP-N67 exhibits chemotaxis toward the GFP-TR4 pathogen. N67-RFP in 9% physiological saline solution was injected into the leaf vascular bundles, and the pathogen GFP-TR4 was placed on the injection wound. **(A)** Disease incidence of banana leaves after 7days’ inoculation. The first two pictures show the front side of the leaves, and the last two pictures show the back side of the leaves. Three replicates were used. **(B)** Microscopy of the control treatment, which injected 9% physiological saline solution with no bacteria, inoculated the GFP-TR4 only. **(C)** Representative micrographs of N67-RFP showed significantly chemotropic toward GFP-TR4. BF, bright field; RFP, red fluorescent field; GFP, green fluorescent field; and MERGE, merge image with BF, RFP, and GFP fields. Three replicates were used. **(D)** Chemotaxis ratio of RFP-N67 toward *Foc* TR4. ^*^indicate the significance between treatments at the 0.05 level.

The interactions between beneficial *Bacillus* strains and pathogens are the key to crop disease control. There are few studies on the interactions between biocontrol bacteria and TR4 in banana. Therefore, we selected RFP-labeled *Bacillus* RFP-N67 and used available green fluorescent labeled pathogens GFP-TR4, inoculating banana leaves simultaneously to observe whether they will interact *in vivo*. Leaf phenotypic observation showed that the lesion size in both the front and back sides of the leaves after the treatment inoculated with RFP-N67 and GFP-TR4 was significantly smaller than those found in leaves inoculated with GFP-TR4 only (Figure 7A). Then, we made ultrathin sections around the leaf lesions and successfully observed RFP-N67 and GFP-TR4 simultaneously under laser confocal microscopy, finding that RFP-N67 often appeared around GFP-TR4 mycelia (Figures 7B,C), and further chemotaxis assay, indicating that RFP-N67 exhibits a strong chemotaxis toward *Foc* TR4 (Figure 7D). Therefore, we can speculate that the biocontrol bacteria RFP-N67 we used can grow and reproduce normally in banana to exert their biocontrol functions, and that in the presence of pathogens, it can be quickly found to inhibit pathogen growth. However, this study only provides histological evidence. Quantitative analysis of specific pathogen growth inhibition needs to be carried out.

## Discussion

Over the last decade, many studies have focused on the interactions of *Foc* with beneficial microorganisms. Generally, researches have centered around “growth promotion,” “systemic resistance induction,” “secondary metabolites syntheses,” etc., which were facilitated by the rapid development of “-omics” tools (Bubici et al., 2019). However, as we know, there are no reliable currently available fluorescent labeled *Bacillus* strains for Banana–Endophyte–Pathogen tritrophic interaction studies. Currently, fluorescence labeling is the best approach for exploring interactions of *Bacillus* spp. with plants *in vivo*. Hence, the fluorescence transformation system we constructed is the first step and of great significance to study the interaction mechanisms associated with TR4 and biological control *Bacillus*. In the process of manipulation, the wild-type *Bacillus* generally has a low transformation efficiency, and some inert *Bacillus* strains cannot even be transformed at all, which seriously affects internal mechanism research of its beneficial properties, such as disease prevention, growth promotion, efficient enzyme production, or antibiotics (He, 2014). Currently, the transformation of *Bacillus* mainly includes protoplast transformation, electric shock transformation, natural transformation, and protoplast electric shock transformation (Shen et al., 2013; He, 2014; Kang, 2019; Plucker et al., 2021; Wang, 2021). In this study, we have explored the different methods and conditions of fluorescent transformation of TR4-antagonistic strains. The results showed that we only got the positive transformants by the method of electric shock transformation. Through the natural transformation method, all seven *Bacillus* strains could not form naturally competent cells or positive transformants, even if we tested in the different transformation conditions, such as culture time, plasmid concentration, and recovery time. The low electro-transformation efficiency of wild-type *Bacillus* may be related to the restriction-repair system in the cell (Zhang et al., 2012). According to the statistics of REBASE (a professional database of restriction endonucleases), approximately 88% bacterial genomes contain restriction-repair systems, and 43% contain four or more restriction-repair systems (Roberts et al., 2007). The restriction-repair system is a barrier for bacteria to exclude external DNA, thus preventing the transformation of bacteriophages and external plasmids, thereby maintaining the integrity and functional stability of its own genetic material. At present, the restriction-repair systems have been found in a variety of bacteria and archaea (Roberts et al., 2003). Finding a way to prevent the wild-type bacteria from degrading the external DNA during the transformation process is the key to improving the efficiency of electric shock transformation.

In the process of cell-wall synthesis, glycine can replace D(L)-alanine in the peptidoglycan component of the bacterial cell wall, which reduces the degree of cross-linking of peptidoglycan and interferes with the synthesis and assembly of the cell wall, thus loosening the cell wall (Anderson et al., 1966; Zhu et al., 2020). It has been reported that the addition of some compounds that inhibit cell-wall synthesis [such as glycine, threonine, penicillin, or Tween 80 (affecting cell membranes’ fluidity)] during the exponential growth stage of *Bacillus* can improve the transformation efficiency (Holo and Nes, 1989; Zhang et al., 2011). After numerous repeated experiments, we added glycine, sorbitol, threonine, and Tween 80 to the electro-transformation growth medium, finding this helps *Bacillus* more easily absorb external DNA to form positive transformants. Although transformation efficiency is between 1×10^2^–10^3^cfu/μg of plasmid DNA in the repetitive experiment, indicating our transformation system is still needed to be further optimized, which means that there is still much space for improvement in the transformation of inert *Bacillus* strains. However, this electric shock transformation system is reproducible and stable because we have generated RFP-labeled *Bacillus* in all seven strains from two main banana producing areas, Yunnan and Guangxi provinces, China (Figure 2B; Supplementary Figures S2A–E).

There are other methods that can also improve transformation efficiency: a good way to temporarily inactivate restriction endonuclease in the host bacterium is by incubating at a certain temperature for a couple of times after electric shock. *Corynebacterium glutamicum* was cultured by shaking under low-temperature to prepare electro-transformation competent cells, and then rapidly heat shocked after the electric shock, the results showed that the electro-transformation efficiency was increased fourfold (Rest et al., 1999). Heat-shocking after electric shock transformation of *B. amyloliquefaciens*, increased transformation efficiency by 10-fold (Zhang et al., 2011). In addition, due to the restriction endonuclease and methylase in the wild-type bacteria restriction-repair systems often appearing in pairs, the *in vivo* methylation modification of the exogenous plasmid to be transformed in the same cell can evade restriction-enzyme digestion and degradation of the host wild-type bacteria during the transformation process, thereby improving the transformation efficiency. Through this strategy, the transformation efficiency of *Bifidobacterium adolescentis* ATCC15703 was increased fivefold (Yasui et al., 2009), and the transformation efficiency of *B. amyloliquefaciens* TA208 and B. cereus ATCC10987 was increased almost fourfold (Zhang et al., 2012).

A vital factor affecting the expression of fluorescent proteins in *Bacillus* is plasmid instability. In order to explore the stability of plasmid pYP69 in *Bacillus* strains, we detected RFP expression and the loss-ratio of plasmid in the transformed strains with regularly sampling. This showed that within 50h of culture, more than 98% of the cells expressed RFP, indicating that very few cells lost their plasmids (Figure 3), and *Bacillus* growth rate was not affected (Figure 4; Supplementary Figures S3A–E). According to previous reports (Bonfrate et al., 2013), the propagation rate of *Bacillus* in natural environments is 50–100h/generation, and the rate of propagation under laboratory conditions is 20–30min/generation. Based on this, it could be calculated that the number of fluorescent strain propagations during 50h is about 100–150 generations. In other words, the plasmid pYP69 still exists stably after 100–150 generations, indicating that it has a very strong compatibility with bacteria. Therefore, RFP-labeled *Bacillus* will be suitable for monitoring its colonizing activities in banana host plants in our next research step.

Nowadays, the biological control of banana Fusarium wilt is always focused on screening antagonistic strains, while ignoring the research on the colonization of antagonistic bacteria in soil or plants. Many of the selected antagonistic bacteria have obvious antibacterial effects *in vitro* or pot experiments, but they usually lose biocontrol effects in the field (Jing et al., 2020; Wei et al., 2020). Therefore, the successful colonization of biocontrol bacteria is an important pre-condition for its application and function in field. Some studies have shown that the colonization of plant growth promoting rhizobacteria (PGPR) is affected by abiotic factors, such as soil temperature, texture, water content, and oxygen content. Biotic factors including root exudates, plant growth conditions, bacterial chemotaxis, the nature of self-regulation mechanisms, and bacterial trophic type also affect colonization (Yajing, 2018). At present, a few studies have been carried out on the colonization of strains controlling banana wilt. Dai (Yajing, 2018) detected the quantity of different PGPR in banana soil rhizosphere by fluorescence quantitative PCR. The results indicated that the biomass of three antagonistic bacteria in banana rhizosphere soil was increased significantly, indicating that the three PGPR strains M8, C5, C14 can colonize banana roots (Lin, 2011). Their colonization determination studies used antibiotic-labeled strains and showed that labeled strains could be isolated by wound inoculation, irrigated inoculation, and axil inoculation. The control did not show any bacterial colonies, indicating that with three methods of inoculation, the labeled strains can be colonized in banana. Chao et al. (2010) used the scanning electron microscope to observe biocontrol bacteria FJAT-346-PA-K in tissue-cultured banana plantlets 10days after inoculation. They successfully found that there were biocontrol bacteria in the roots’ internal tissues and banana stems. We inoculated the wild-type strain N67 into tissue-cultured banana plantlets, and it was also confirmed by scanning electron microscope that N67 could successfully colonize the banana root cells (Figures 6B,C). However, this method can only preliminarily judge the existence of biocontrol strains in the plant, and it cannot clearly figure out the internal activity *in vivo*. Therefore, we constructed red fluorescent-labeled strains to observe its dynamic migration in banana plants, and the results showed that the biocontrol strains can successfully colonize the roots but not in the corm or pseudostem due to short incubation period (Figure 6A). We also injected RFP-labeled bacteria into the detached leaf which is proved to be a reliable protocol (Liu et al., 2020). The results showed that the biocontrol strains can successfully colonize and grow in the leaf, as well as displaying a positive chemotaxis response toward TR4 hyphae, indicating that the biocontrol bacteria can effectively interact with and inhibit the pathogen *in vivo* (Figure 7). Other studies have shown that chemoattraction of bacteria could contribute to its root colonization (Palmieri et al., 2020). *Fusarium oxysporum* f. sp. *lycopersici* (*Fol*) is known to facilitate bacterial movement in search of nutrients which also exhibits chemotaxis toward plant roots (Furuno et al., 2009; Turra et al., 2015). We thus conclude that N67 not only directly inhibits TR4 hyphae growth, but it could also benefit from the capacity of pathogen hyphae trends to plant colonization and thus increases colonization efficiency. Significant progress has been made in other crops by using fluorescent-labeled strains for colonization observation. Kang (2019) observed that the endophytic *B. velezensis* CC09 *Bv*-GFP can not only effectively colonize wheat roots, but also migrate to stem and leaf organs to achieve whole plant distribution. He (2014) observed the roots of maize seedlings inoculated with Y2-P43GFPmut3a by fluorescence microscope, showing that Y2 strain successfully colonized root surfaces and interiors. However, as far as we know, there is no research on the *in vivo* interaction of RFP-*Bacillus* for monitoring TR4 in banana, so dynamic migration is our next proposed research.

With the great progress of gene-sequencing technology, more and more *Bacillus* whole genomes have been sequenced. Using such tools, it will be important to explore the potential biocontrol mechanisms among *Bacillus*, pathogens, and plants (Bubici et al., 2019; Carrión et al., 2019; Jiang et al., 2019; Chen et al., 2020; Jing et al., 2020; Wei et al., 2020). The biocontrol mechanisms of *Bacillus* are usually considered to be based on one or more of: (1) Antagonism: *Bacillus* often secretes secretory secondary metabolites that inhibit pathogen growth. (2) Competition: *Bacillus* competes for niches with pathogens and other microorganisms to obtain nutrients and other resources. (3) Inducing systemic resistance: they can activate host defense responses by inducing systemic resistance. (4) Promoting growth: *Bacillus* can provide necessary mineral nutrition and plant hormones (e.g., IAA) for plant hosts to support their life activities. These mechanisms of *Bacillus* are interrelated and synergistic. Of course, the principal *Bacillus* biocontrol mechanism(s) could be different in different crops.

It is an innovative research direction to develop new biopesticides and new agricultural antibiotics by using the antagonistic behavior of microorganisms (Yajing, 2018). It has become an important measure for biological control of TR4 in organic farming by researching “biological fertilizer” (where organic fertilizers are inoculated with biocontrol agents) and banana growth-promoting bacterial agents to increase yield and disease resistance (Ling et al., 2014; Fu et al., 2016). Nowadays, biological control for sustainable banana production has attracted much attention. New biological microorganism agents are urgently needed to replace traditional chemical pesticides, which continue to cause pollution and damage the environment, and degrade soils. Microbial agents as recognized promising “pollution-free pesticides” will play a crucial role in managing agricultural and silvicultural diseases and protecting ecological balance.

## Conclusion

We successfully developed an optimized fluorescent electro-transformation system of TR4-inhibitory *Bacillus* spp. strains (OD600=0.7, plasmid concentration=50ng/μl, volume=2μl, voltage=1.8kV, and capacitance=400Ω). The RFP-labeled *Bacillus* strains have high stability, and their growth rates and inhibition effects on TR4 are unaffected by fluorescent plasmid insertion. *In vivo* colonizing observation by Laser Scanning Confocal microscopy (LSCM) and SEM showed that *Bacillus* spp. can colonize the xylem cells of banana plantlets’ roots. Further fluorescent observation by LSCM showed these RFP-labeled bacteria exhibit chemotaxis toward the hyphae of the green fluorescent protein (GFP)-labeled TR4 pathogen in banana leaves.

## Data Availability Statement

The original contributions presented in the study are included in the article/Supplementary Material, further inquiries can be directed to the corresponding authors.

## Author Contributions

PH conceived, designed, and performed the experiment, analyzed the data, and wrote the paper. SL conceived, designed, and performed the experiment, and analyzed the data. S-JZ conceived and designed the experiment and prepared the manuscript. SX, HF, YW, and GH analyzed the data. WZ and GF provided the strains sources and analyzed the data. S-JZ and Y-YW supervised the research and provided funding support. All authors contributed to the article and approved the submitted version.

## Funding

This research was funded by Science and Technology Department of Yunnan Provincial Government (202001AU070150 and 202102AE090003), Yunling Scholar Programme of Yunnan Provincial Government (YNWR-YLXZ-2018-018), Program for the Innovative Research Team of Green Prevention and Control of Agricultural Transboundary Pests of Yunnan Province (2020–2022), the CGIAR Re-search Program (CRP) on Roots, Tubers, and Bananas (RTB). CRPs are implemented with support from the CGIAR Trust Fund and through bilateral funding agreements. For details, please visit https://ccafs.cgiar.org/donors. The views expressed in this document cannot be taken to reflect the official opinions of these organizations.

## Conflict of Interest

The authors declare that the research was conducted in the absence of any commercial or financial relationships that could be construed as a potential conflict of interest.

## Publisher’s Note

All claims expressed in this article are solely those of the authors and do not necessarily represent those of their affiliated organizations, or those of the publisher, the editors and the reviewers. Any product that may be evaluated in this article, or claim that may be made by its manufacturer, is not guaranteed or endorsed by the publisher.
