# Supplementary material for: Monitoring Tritrophic Biocontrol Interactions Between Bacillus spp., Fusarium oxysporum f. sp. cubense, Tropical Race 4, and Banana Plants in vivo Based on Fluorescent Transformation System
Source: Front Microbiol. 2021 Oct 13;12:754918. doi: 10.3389/fmicb.2021.754918 (PMC8550332; doi:10.3389/fmicb.2021.754918)
Supplement: Supplementary file 1 [file Table_1.DOCX]

Supplementary Material

# Supplementary Figures


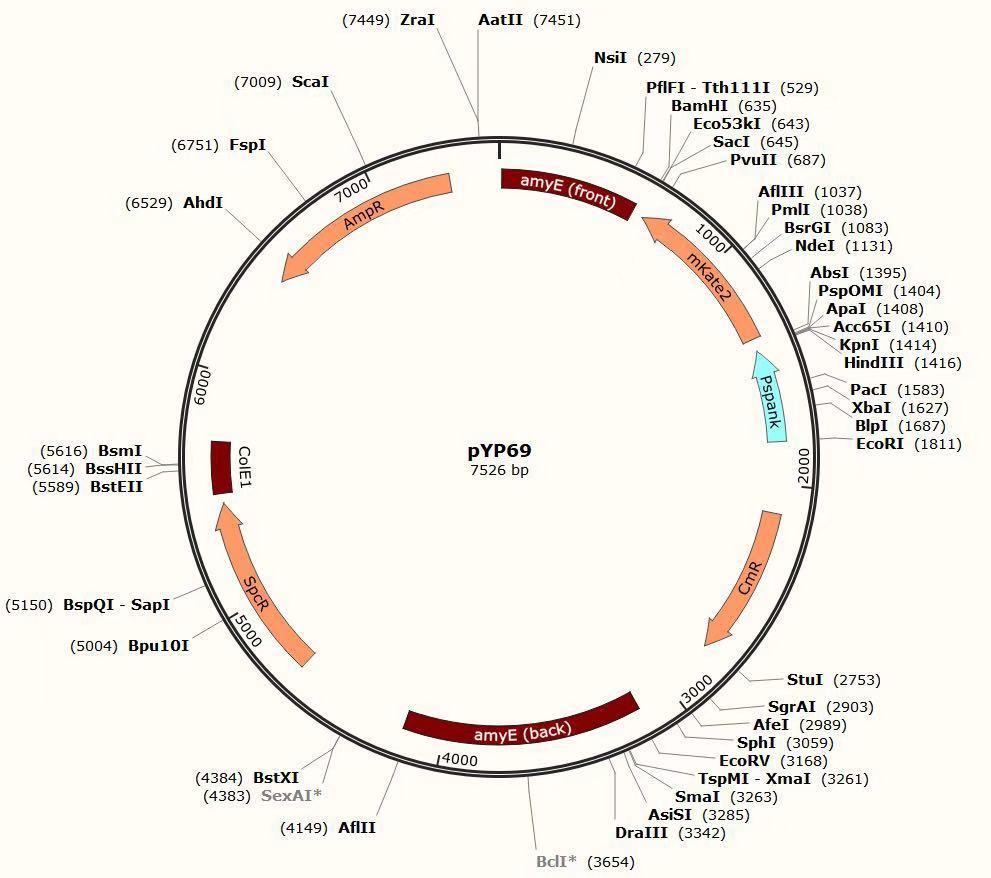


**Supplementary Figure S1.** The genetic map of plasmid pYP69. The plasmid expressing RFP (mKate2) and the chloramphenicol-resistance gene. mKate2 coding sequence with ribosome binding site sequence was cloned by PCR using genomic DNA of YC843 as template. The PCR product was digested with HindⅢ and BamHI, ligated with pYC127 digested with the same restriction enzymes. This plasmid is used to make over expression RFP strain in *Bacillus*.


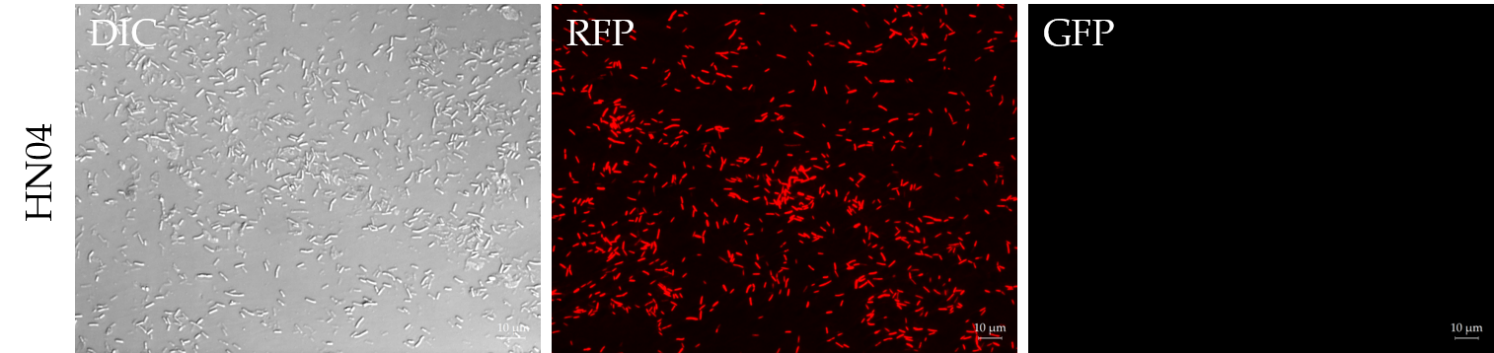


**(a)**


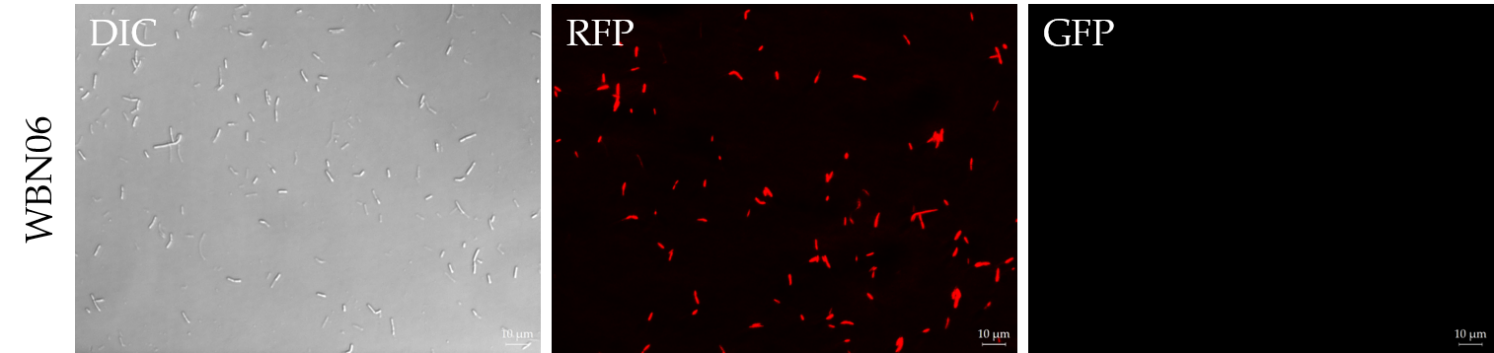


**(b)**


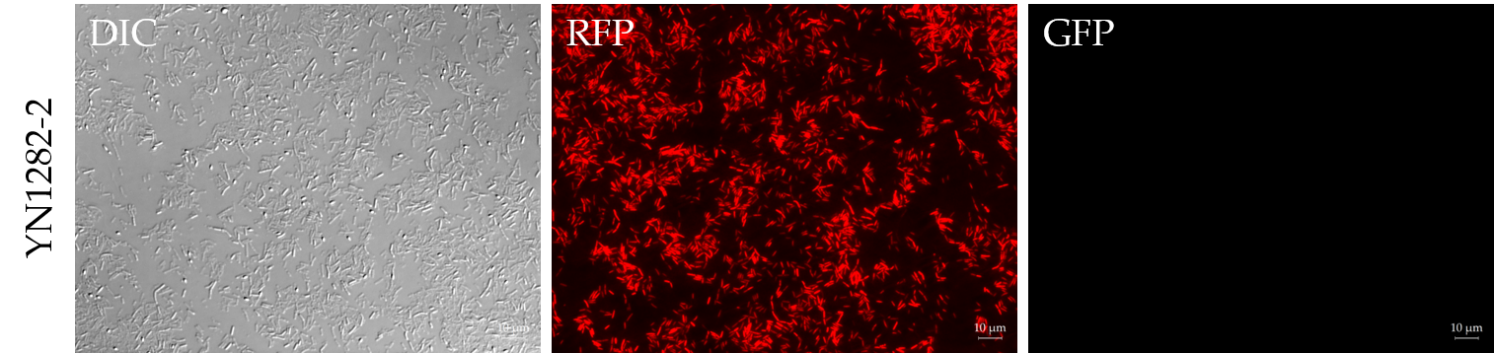


**(c)**


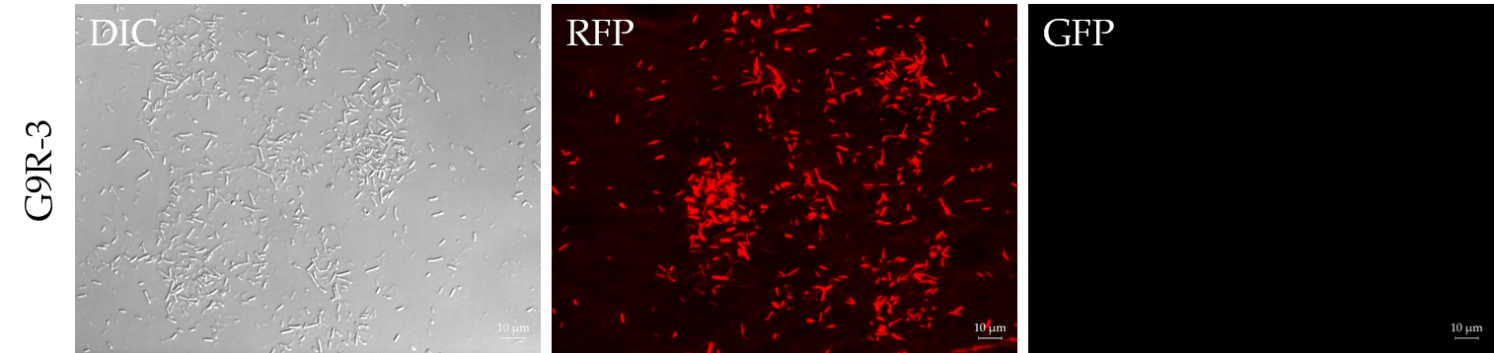


**(d)**


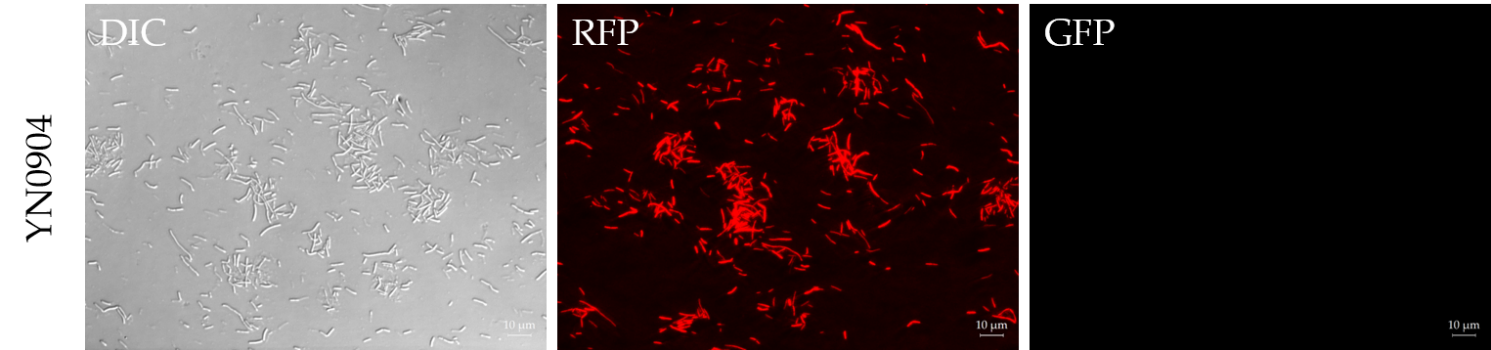


**(e)**


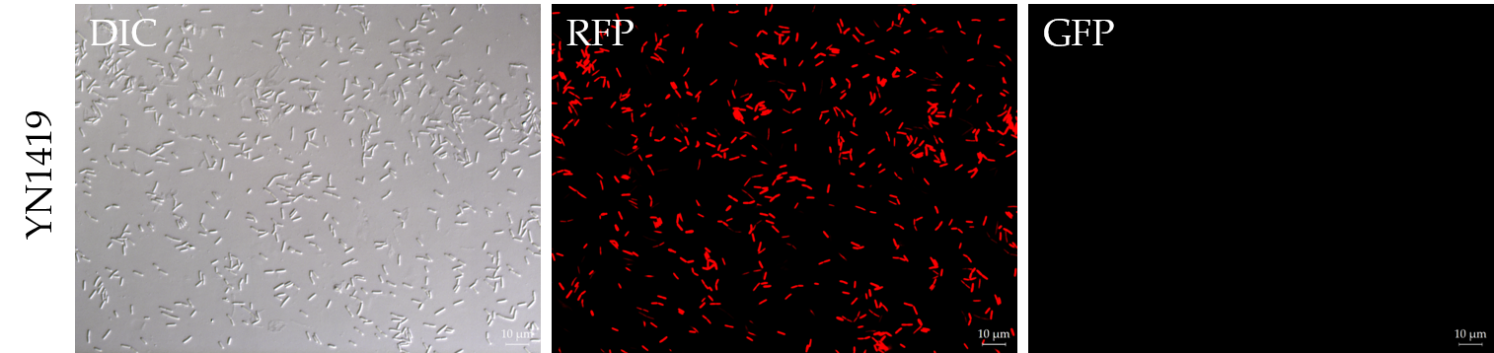


**(f)**

**Supplementary Figure S2.** Fluorescence observation of RFP-*Bacillus* under microscopy. DIC, differential interference contrast field; RFP, red fluorescence field; GFP, green fluorescence field. Electric-transformation was used to transform pYP69 into *Bacillus*. Positive transformants on the solid medium containing 10 μg/mL chloramphenicol could be observed using microscopy. (a) Fluorescence observation of RFP-HN04 under microscopy. (b) Fluorescence observation of RFP-WBN06 under microscopy. (c) Fluorescence observation of RFP-YN1282-2 under microscopy. (d) Fluorescence observation of RFP-G9R-3 under microscopy. (e) Fluorescence observation of RFP-YN0904 under microscopy. (f) Fluorescence observation of RFP-YN1419 under microscopy. Scale bar: 10 μm.















**Supplementary Figure S3.** The growth rate of RFP-*Bacillus* compared to the wild-type. OD600 measures were used to determine bacterial growth rate. (a) The growth rate of RFP-HN04 compared to the wild-type. (b) The growth rate of RFP-WBN06 compared to the wild-type. (c) The growth rate of RFP-YN1282-2 compared to the wild-type. (d) The growth rate of RFP-G9R-3 compared to the wild-type. (e) The growth rate of RFP-YN0904 compared to the wild-type. (f) The growth rate of RFP-YN1419 compared to the wild-type. Three replicates were included.

**Supplementary Table S1.** **The inhibition effects of RFP-*Bacillus* compared to the wild-type.**

| Strains | | Inhibition rate (%) | | | | | Significance | |
| --- | --- | --- | --- | --- | --- | --- | --- | --- |
|  |  | replicate 1 | replicate 2 | replicate 3 | Average | Standard error |  |  |
| N67 | WT | 50.88 | 48.28 | 57.58 | 52.24 | 2.77 | a | ns |
|  | RFP | 47.37 | 51.72 | 54.55 | 51.21 | 2.09 | a |  |
| HN04 | WT | 49.21 | 46.88 | 48.48 | 48.19 | 0.69 | a | ns |
|  | RFP | 42.86 | 46.88 | 45.45 | 45.06 | 1.18 | a |  |
| WBN06 | WT | 49.15 | 49.15 | 51.61 | 49.97 | 0.82 | a | ns |
|  | RFP | 49.15 | 45.76 | 51.61 | 48.84 | 1.70 | a |  |
| YN1282-2 | WT | 58.62 | 61.29 | 57.38 | 59.10 | 1.15 | a | ns |
|  | RFP | 51.72 | 58.06 | 60.66 | 56.81 | 2.65 | a |  |
| G9R-3 | WT | 52.73 | 49.10 | 56.67 | 52.83 | 2.19 | a | ns |
|  | RFP | 49.10 | 49.10 | 56.67 | 51.62 | 2.53 | a |  |
| YN0904 | WT | 54.39 | 50.88 | 53.33 | 52.87 | 1.04 | a | ns |
|  | RFP | 47.37 | 47.37 | 53.33 | 49.36 | 1.99 | a |  |
| YN1419 | WT | 60.56 | 53.13 | 56.25 | 56.65 | 2.16 | a | ns |
|  | RFP | 60.56 | 53.13 | 53.13 | 55.60 | 2.48 | a |  |

Data with different lowercase letters indicate a significant difference at the 0.05 level. ‘ns’ indicates there are no significant difference between the two strains at the 0.05 level.
